# Augmentation Index in Patients with Thoracic Aortic Aneurysm: A Matched Case-Control Study

**DOI:** 10.3390/jcdd10010006

**Published:** 2022-12-23

**Authors:** Patrick Baumgartner, Protazy Rejmer, Martin Osswald, Stefan Malesevic, Noriane A. Sievi, Maurice Roeder, Jonas Herth, Simon F. Stämpfli, Christian F. Clarenbach, Felix C. Tanner, Thomas Gaisl, Malcolm Kohler

**Affiliations:** Department of Pulmonology, University Hospital Zurich (USZ), University of Zurich (UZH), 8091 Zurich, Switzerland

**Keywords:** thoracic aortic aneurysm, augmentation index, arterial stiffness

## Abstract

Thoracic aortic aneurysms (TAA) may be associated with complications such as rupture and dissection, which can lead to a fatal outcome. Increased central arterial stiffness has been proposed to be present in patients with TAA compared to unmatched controls. We aimed to assess whether wall properties in patients with TAA are also altered when compared to a matched control group. Applanation tonometry was performed in 74 adults with TAA and 74 sex, age, weight, height, and left ventricular ejection fraction matched controls. Subsequently analysis of the pulse wave was done using the SphygmoCor System. For comparing the two groups, AIx was adjusted to a heart rate of 75/min (AIx@75). 148 1-to-1 matched participants were included in the final model. There was no significant difference in the Alx@75 between the TAA group and the matched control group [mean (SD) of 24.7 (11.2) % and 22.8 (11.2) %, *p* = 0.240]. Adjusted for known cardiovascular risk factors, there was no association between TAA and AIx@75. Patients with TAA showed comparable arterial wall properties to cardiovascular risk factor matched controls. Since higher arterial stiffness is associated with TAA progression, it remains to be investigated if increased central arterial stiffness is a relevant factor of TAA emergence.

## 1. Introduction

A thoracic aortic aneurysm (TAA) is a localized dilation of the aorta in the thorax. TAA have an incidence rate of approximately 10 per 100,000 persons-years in both sexes [1]. Generally, TAA occur sporadic and in association with risk factors for atherosclerosis such as arterial hypertension, smoking, dyslipidemia and inherited connective tissue disease [2,3]. Most of the TAA do not cause any symptoms and are therefore clinically silent. Nevertheless, they are associated with life threating complications such as dissections and ruptures. The risk of rupture mainly depends on the diameter of the aneurysm and the mean rate of rupture is estimated to be 2–3% per year [4,5]. The formation of TAA is usually caused by cystic media degeneration. In this process, the elastic fibers in the aortic wall degenerate und cause weakening of the wall leading to a dilatation of the aorta. The process of cystic media degeneration is also associated with aging and conventional cardiovascular risk factors [6]. Increased aortic stiffness and its adverse effects on pressure buffering are intensively described in the literature. Damage to the aortic wall disrupts its mechanical properties, stiffening the wall and impairing its pressure buffering function, which increases pulsatile arterial load [7]. Augmentation index (AIx) has been proposed as a measure of aortic stiffness and wave reflection [8]. TAA and increased arterial stiffness share risk factors, such as age, obesity, dyslipidemia and arterial hypertension [9,10]. Additionally, chronic inflammation and changes in structural elements of the arterial wall, in vascular smooth muscle tone and endothelial dysfunction are associated with increased arterial stiffness [11,12]. Altered aortic wall structure, dedifferentiated smooth muscle cells, endothelial dysfunction as well as inflammation has been shown to be associated with TAA formation or progression [13,14,15,16]. Changes in the arterial wall are therefore associated with both the pathogenesis of TAA and arterial stiffness. Previous studies showed an increased AIx in patients with TAA and abdominal aortic aneurysm (AAA) compared to an unmatched control group [17,18]. Furthermore, studies showed that there is a linear association between central arterial stiffness and TAA progression [19,20]. However, it remains unknown if patients with TAA altered arterial wall properties when compared with a sex, age, weight, height, and left ventricular ejection fraction (LVEF) matched control group. 

## 2. Materials and Methods

### 2.1. Study Design and Participants

This study is a secondary analysis of data from two previously published and independent studies. Patients with TAA recruited between July 2014 and March 2020 in a previously published study that assessed the progression of aortic aneurysm in patients with obstructive sleep apnea [21]. TAA was defined as an aortic diameter higher than sex-specific cut-offs at the level of the sinus of Valsalva (≥39 mm for women, ≥44 mm for men) or at the level of the ascending aorta (≥42 mm for women, ≥46 mm for men) [22]. The study included a short interview about medical history and a 3-year follow-up with yearly echocardiography. Arterial applanation tonometry at the level of the radial artery using the SphygmoCor System (AtCor Medical, Sydney, NSW, Australia) was performed at the last visit. The control group of this study was part of a previously published study, which investigated the prevalence of obstructive sleep apnea in patients with TAA [23]. In this former study, 208 patients with a TAA were 2-to-1 matched with 104 participants without a dilatation of the aorta. The matched control group was recruited between January and November 2018 and underwent a short medical interview and an arterial applanation tonometry. Out of the two aforementioned studies, patients with TAA and 1-to-1 matched controls were analysed. The participants for this study were matched for sex (exact), age (±5 years), weight (±20 kg), height (±20 cm) and left ventricular ejection fraction (LVEF) (±10%). 

### 2.2. Echocardiography

The measurement of the diameter of the aorta was performed using a cardiovascular ultrasound system with a 3.5 MHz transducer (Vivid E9 with XDclear, GE Healthcare, Little Chalfont, UK). Along the left parasternal long axis window, the diameter of the aortic sinus, defined as the largest diameter at the sinus of Valsalva and the ascending aorta at the level of the right pulmonary artery was measured. The measurements were assessed by a two-dimensional, M-Mode guided echocardiography in endsystole. Every measurement was done three times in a row and the mean out of this measurement was calculated. The LVEF was calculated using the biplane Simpson’s method [24]. As recommended, the leading edge-to-leading edge technique was used for the measurements in the study [25].

### 2.3. Blood Pressure Measurement

Blood pressure was measured in a sitting position, after a resting time of 5 min. It was measured using a standard digital automatic monitor (Omron Healthcare Company, Kyoto, Japan) three times in a row on both arms. The mean systolic blood pressure values of both arms were calculated and the values of the arm with the higher mean systolic blood pressure were used for further analysis [26].

### 2.4. Pulse Wave Analysis

**Figure 1:**
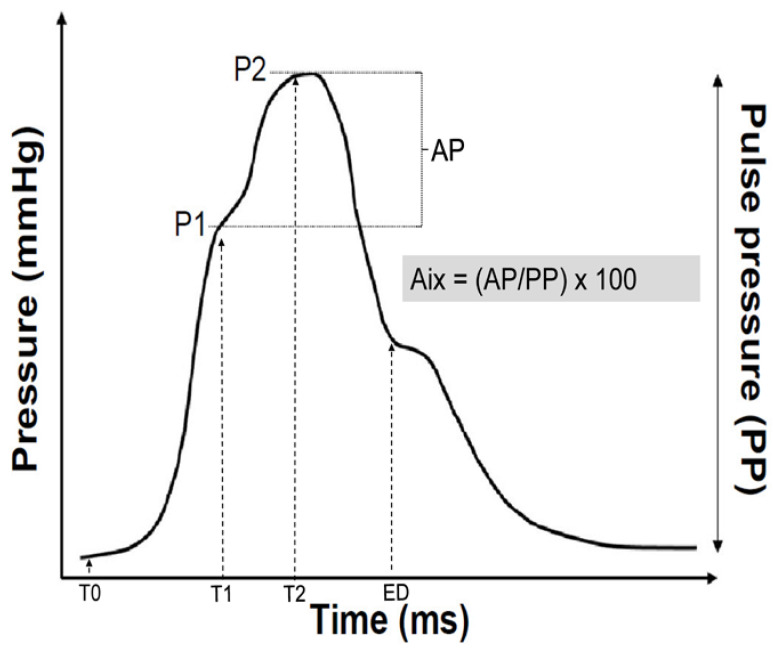
Example of a synthesized aortic pulse pressure wave derived from form analysis of the recorded radial artery pulse wave. After the start of the pulse wave (T0), indicating the onset of ejection, the pressure wave rises to an initial peak where it forms a shoulder (P1). This is the peak of the primary left ventricular ejection pressure. The second shoulder (P2) represents the peak of the arterial reflection wave. The difference between P2 and P1 is called augmentation pressure (AP). The end of ejection (ED) is the point of closure of the aortic valve and time of the end of systole. The augmentation index (AIx) is calculated as the difference between second (P2) and first (P1) systolic peak pressure.

Every participant underwent a radial pulse wave analysis using the SphygmoCor System (AtCor Medical, Sydney, NSW, Australia). Radial artery applanation tonometry is a valid technique to easily assess pulse wave recording [27]. Subjects were asked to abstain from caffeine, alcohol and tobacco on the day of measurement. The subjects were asked to only take a light meal before the measurement. Mean brachial systolic and diastolic blood pressure values of the arm with the higher were used to calibrate the radial pulse wave analysis. After blood pressure measurement, subjects had to rest 10 min in a quiet room before the pulse wave analysis took place in sitting position. Between 7 to 10 waves were recorded before the analysis. For high measurement quality, only measurements with an operator index of 80 or higher were used for final analysis. Measurements were performed by two trained and very experienced operators. SphygmoCor uses a validated mathematical function to generate a central aortic pulse wave and thus aortic augmentation index [28]. The inflection point of the aortic pulse wave curve marks the moment of the arrival of the reflected pulse wave in the ascending aorta. The contribution of the reflected wave to the systolic arterial pressure is named pressure augmentation (AP). Alx can be calculated as the as the difference between the systolic blood pressure and the pressure at the inflection point (=AP) divided by the central pulse pressure (PP). AIx is therefore expressed as percentage of the central pulse pressure (Figure 1).

AIx (%) = (AP/PP) × 100%

Since AIx is influenced by heart rate, the index is adjusted to a heart rate of 75 beats per minute (AIx@75) [29]. 

### 2.5. Statistical Analysis

Statistical analysis was done using STATA Version 16 (StataCorp LP, College Station, TX, USA). Continuous results are expressed as mean ± standard deviation (SD) or median (interquartile range) as appropriate. The groups were compared using paired t-test or Wilcoxon signed rank test for continuous variables. A two sided *p*-value of <0.05 was considered significant. For the analysis of the independent association between AIx and TAA, multivariable analysis was used with adjustment of pre-specified, known cardiovascular risk factors such as blood pressure, smoking, diabetes and use of medication [30,31,32,33,34,35,36]. Residual analysis of the model was performed to check the regression assumptions.

### 2.6. Statement of Ethics

All participants were informed verbally and in writing about the study. They had to give their written informed consent prior to participation in the study. The study and procedures were approved by the Cantonal Ethics Committee Zurich, Switzerland (KEK-ZH-Nr. 2014-0035). The study and procedures were done in conformity with the Declaration of Helsinki. Upon reasonable request, data supporting the findings of this study are available.

## 3. Results

### 3.1. Patient Characteristics

**Figure 2:**
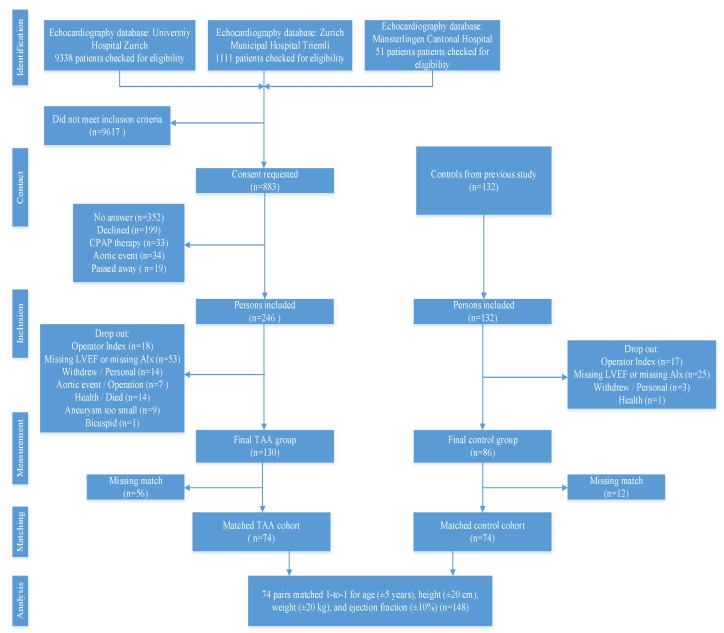
All TAA patients were enrolled from echocardiography databases of three different hospitals. Patients from the University Hospital Zurich accounted for the most of the included persons. Final analysis consisted of 148 participants, one-to-one matched. CPAP: continuous positive airway pressure, LVEF: left ventricular ejection fraction, AIx: augmentation index, TAA: thoracic aortic aneurysm.

The cohort of TAA patients consisted of 246 patients. 53 patients were excluded due to the lack of LVEF values or missing SphygmoCor data. Due to low operator index, a further 18 patients in the TAA group had to be excluded. The cohort of controls consisted of 132 patients with the largest part of excluded patients also due to a lack of LVEF or missing SphygmoCor data. For more details, see the study flow chart (Figure 2). 56 patients of the TAA group and 12 of the control group could not be matched thus resulting in 74 patients from the TAA cohort and 74 (1-to-1 matched) patients from the control cohort in the final analysis. The median (IQR) age in the TAA group was 68 (60/75) years and 68 (59/73) years for the control group, respectively. Of the matched patient characteristics, systolic and diastolic blood pressure were higher in TAA patients compared to control subjects (mean (SD) of 130.2 ± 15.1 mmHg and 122.0 ± 12.6 mmHg for systolic blood pressure, 80.7 ± 9.7 mmHg and 77.1 ± 9.2 mmHg for diastolic blood pressure, respectively). Arterial hypertension had a higher prevalence in the TAA group compared to the control group (73% vs. 50%). The prevalence of diagnosed diabetes mellitus was comparable between groups. In terms of medication use, the difference was relevant for antihypertensive drugs (81% of TAA patients vs. 61% of controls) and for betablockers (BBs) (47% in TAA vs. 31% in control). In terms of statins, diuretic drugs, new oral anticoagulants, and antiplatelet drugs there was no obvious difference between these two groups (Table 1, not all data shown).

### 3.2. Pulse Wave Analysis

**Figure 3:**
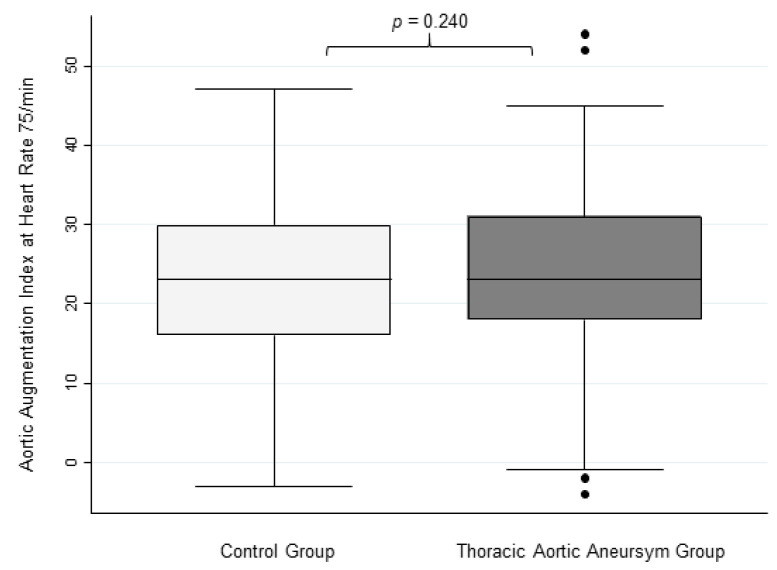
Aortic Augmentation Index at Heart Rate 75/min. This figure shows box plots for aortic augmentation index adjusted to a heart rate of 75/min in controls and TAA patients with no significant differences.

Although the unadjusted aortic AIx showed a significant difference between the TAA cohort and control subjects, TAA patients showed no significant difference in aortic AIx@75 compared with the control cohort (24.7 ± 11.2% vs. 22.8 ± 11.2%, respectively, Figure 3). 

Regarding peripheral AIx, there was also no significant difference between the two groups. There was no significant difference between the groups in the primary wave pressure at T1. Looking at heart parameters, the resting heart rate in the TAA cohort was slower, the ejection duration on the other hand was significantly shorter in the control group (Table 2).

An additional analysis of AIx@75 was performed in a subgroup of sex, age and blood pressure matched controls (50 TAA patients with 1-to-1 matched 50 controls, data not shown). There was no significant difference in AIx@75 between TAA and these matched controls.

### 3.3. Association between Aortic AIx and TAA Adjusted for Known Cardiovascular Risk Factors

There was no evidence for an association between AIx@75 and the presence of TAA using a non-adjusted regression model (Coef. (95% CI) of 1.07 (−2.58 to 4.73), *p* = 0.562). Adjusted for known cardiovascular risk factors and use of medication (Table 3), AIx@75 showed no association with the presence of TAA (Coef. (95% CI) of 0.58 (−3.44 to 4.60), *p* = 0.777).

## 4. Discussion

To our knowledge, this is the first study to compare aortic augmentation indices between patients with thoracic aortic aneurysm and a matched control group. Patients with TAA showed a comparable AIx@75 compared with a sex, age, height, weight, and LVEF matched control subjects. This implies no relevantly altered arterial wall properties in TAA patients despite the known fact that increased arterial wall properties prone TAA progression.

Most of TAA occur sporadic but still in association with classical risk factors for atherosclerosis. However, these risk factors poorly predict the incidence of TAA, suggesting additional pathomechanisms [2]. Possible explanations have been discussed in the literature. Pisano et al. [14] discussed the role of inflammation in TAA pathogenesis. Other studies assessed the influence of dedifferentiated smooth muscle cells and endothelial dysfunction in the pathogenesis of TAA [13,15,16]. Moreover, these pathomechanisms have been shown to be associated with increased arterial stiffness [11,12].

Shingu et al. [18] showed an increased AIx in patients with TAA and chronic aortic dissection compared to a control group. In 21 patients with TAA, arterial diameter changes at level of carotid artery were measured, using an echo-tracking system. Although the study showed an increased AIx in patients with TAA compared to controls, there are some relevant limitations to the study. Beside the small number of participants, the control group was not matched in terms of sex, age, and LVEF. This seems to be important since previous studies reported associations between AIx and left ventricular contractility, and an influence of sex on augmentation index [37,38]. Besides that, AIx was not adjusted for heart rate, which is known to influence AIx [29]. Addressing these limitations in our matched control study, we were not able to confirm the findings of Shingu et al. [18]. In our study, coronary heart diseases (CHD) were more prevalent in the control group compared to the TAA group, therefore suggesting a difference in risk factors for cardiovascular diseases [39]. To counteract this possible influence on AIx, we adjusted for known cardiovascular risk factors and CHD [40]. After adjusting for known cardiovascular risk factors, there was no evidence for an influence of TAA on altered arterial wall properties. Elevated arterial stiffness has also been shown in patients with AAA [17]. Durmus et al. [17] conducted a study using the SphygmoCor system. They showed an increased AIx in patients with AAA. The study consisted of 18 patients with AAA and 20 age and sex matched controls. Again, this study is limited due to the small patient number. Further, vasodilatating drugs such as angiotensin-converting enzyme inhibitor (ACEI) were used more frequently in the control group and not adjusted for in the analysis. ACEI and vasodilatating drugs can decrease AIx [41] and therefore could have increased the difference between groups.

Arterial hypertension has been proposed to be the greatest population-attributable risk factor leading to TAA [42,43,44]. Our study supports this previous finding since there is a significant difference in use of antihypertensive medication between the two groups and therefore implies that arterial hypertension is more frequently seen in patients with TAA. However, adjusting for this imbalance between groups in a subgroup analysis revealed no influence of TAA on AIx in our study.

To date, progression rates of thoracic aortic are poorly understood. Main factors leading to TAA growth are aneurysm diameter, the anatomical location, presence of Marfan-Syndrome, or bicuspid aortic valve [45]. In addition, hyperlipidemia has also been reported as a cofactor influencing the expansion rate [46]. Few additional studies assessed the influence of arterial stiffness on the progression of TAA. Recently, Boczar et al. [19] conducted a study in TAA patients assessing determinants of future TAA expansion. They showed that central systolic blood pressure and increased arterial stiffness, measured by combining arterial tonometry and echocardiography, are independently associated with TAA progression. Zhu et al. [20] recently showed an improvement in TAA progression prediction combining aortic stiffness and aneurysma size at baseline. Thus, both studies highlight a correlation between TAA progression and aortic stiffness despite mean pulse wave velocity (PVW) were within normal ranges (9.65 ± 3.66 m/s and 9.24 ± 3.35 m/s) in the TAA patients in both studies. Taking all these findings into account we assume arterial wall properties are not primarily increased in TAA patients but as larger the alteration of arterial wall properties as greater the risk for TAA progression.

A limitation is the relevant difference in use of medication between groups. As expected, there were more patients in thoracic aortic aneurysm cohort using betablockers and antihypertensive drugs. Betablockers can influence the aortic augmentation index through reducing the heart rate and thus enhance AIx [47] while vasodilatating drugs can influence AIx by decreasing it [41]. To take this into account a normalized Aix for a heart rate of 75 bpm was used and the final model was adjusted for the use of antihypertensive drugs. The study was not powered for this analysis. However, minimal important difference in AIx seems to be around 6.7% [48], indicating a sample size of 3 participants per group. Therefore, a relevant type 2 error seems not to dominate.

Further studies assessing the impact of arterial stiffness on TAA emergence, progression and cardiovascular events with more specific methods are needed, since the lack of larger altered arterial wall properties in TAA patients does not exclude a possible effect of altered arterial wall properties on TAA.

## 5. Conclusions

Our study showed no difference in heart rate adjusted aortic augmentation index between a TAA cohort and a matched control group, therefore suggesting comparable arterial wall properties. However, wheather increased arterial stiffness favor the emergence of TAAs needs to be evaluated in further studies.

## Figures and Tables

**Table 1:** Baseline characteristics of the one-to-one matched study participants.

	TAA Cohort (n = 74)	Control Group (n = 74)
Sex (f / m)	15 / 59	15 / 59
Age, years	68 (60/75)	68 (59/73)
Height, cm	176.2 ± 9.6	173.2 ± 9.3
Weight, kg	81.0 ± 12.2	79.1 ± 13.5
BMI, kg/m^2^	25.9 (23.5/27.8)	25.7 (23.7/28.4)
BSA, m^2^	2.0 ± 0.2	1.9 ± 0.2
Systolic blood pressure, mmHg	130.2 ± 15.1	122.0 ± 12.6
Diastolic blood pressure, mmHg	80.7 ± 9.7	77.1 ± 9.2
Pulse, bpm	65 (58.3/71.7)	69 (63.7/79)
Smoker, n (%)	7 (9)	13 (18)
Pack years, py	15 (0/30)	5 (0/22)
Diabetes, n (%)	3 (4)	8 (11)
Arterial Hypertension, n (%)	54 (73)	37 (50)
CHD, n (%)	19 (26)	29 (39)
Statin, n (%)	42 (57)	41 (55)
Antidiabetic drugs, n (%)	4 (5)	4 (5)
Antihypertensive drugs, n (%)	60 (81)	45 (61)
Betablockers, n (%)	35 (47)	23 (31)
Antiplatelet drugs, n (%)	35 (47)	34 (46)
Aortic sinuses diameter, mm	44 (41/47)	36 (32/39)
Ascending aortic diameter, mm	44 (40/46)	35(32/38)
Aortic sinueses aneurysm, n (%)	51 (69)	0 (0)
Ascending aortic aneurysm, n (%)	28 (38)	0 (0)

TAA: thoracic aortic aneurysm, BMI: Body-Mass-Index, BSA: body surface area, CHD: coronary heart disease.

**Table 2:** Parameters of the pulse wave analysis for the TAA cohort and the control group.

	TAA Cohort (n = 74)	Control Group (n = 74)	*p*-Value
Aortic Alx (AP/PP) @75bpm, %	24.7 ± 11.2	22.8 ± 11.2	0.240
Augmentation Index (AP/PP) %	29.7 ± 12.7	25.0 ± 12.8	0.020
Peripheral Alx, %	90 (82/105)	90 (78/95)	0.357
P1 height, mmHg	27.3 ± 6.7	25.5 ± 5.9	0.108
Heart rate, bpm	64.0 ± 10.9	70.6 ± 11.3	<0.001
Ejection duration, ms	318.3 ± 26.9	292.2 ± 27.5	<0.001

TAA: thoracic aortic aneurysm, bpm: beats per minute, AIx: augmentation index, AP: augmentation pressure, PP: pulse pressure.

**Table 3:** Multivariable linear regression for association between AIx@75 and TAA adjusted for known cardiovascular risk factors and use of medication (n = 148).

Variable	Coefficient	95% Confidence Interval	*p*-Value
TAA, (yes/no)	0.58	−3.44 to 4.60	0.777
Systolic blood pressure, mmHg	0.03	−0.11 to 0.17	0.642
Arterial hypertension, (yes/no)	3.51	−0.67 to 7.69	0.099
Coronary heart disease, (yes/no)	1.87	−3.05 to 6.79	0.454
Diabetes, (yes/no)	−0.96	−7.90 to 5.97	0.784
Statin, (yes/no)	0.10	−4.53 to 4.74	0.965
Pack years of smoking, n	0.08	−0.10 to 0.18	0.080

TAA: thoracic aortic aneurysm, AIx@75: augmentation index adjusted to a heart rate of 75/min.

## Data Availability

The datasets generated and/or analyzed during the current study are available from the corresponding author upon reasonable request.
